# Identification of breast cancer risk modules via an integrated strategy

**DOI:** 10.18632/aging.102546

**Published:** 2019-12-20

**Authors:** Wan Li, Gui Deng, Ji Zhang, Erqiang Hu, Yuehan He, Junjie Lv, Xilin Sun, Kai Wang, Lina Chen

**Affiliations:** 1 College of Bioinformatics Science and Technology, Harbin Medical University, Harbin, China; 2 Molecular Imaging Research Center (MIRC), Harbin Medical University, Harbin, China; 3 TOF-PET/CT/MR Center, the Fourth Hospital of Harbin Medical University, Harbin, China

**Keywords:** breast cancer, disease risk module, integrated strategy, multi-objective programming model, permutation test

## Abstract

Breast cancer is one of the most common malignant cancers among females worldwide. This complex disease is not caused by a single gene, but resulted from multi-gene interactions, which could be represented by biological networks. Network modules are composed of genes with significant similarities in terms of expression, function and disease association. Therefore, the identification of disease risk modules could contribute to understanding the molecular mechanisms underlying breast cancer. In this paper, an integrated disease risk module identification strategy was proposed according to a multi-objective programming model for two similarity criteria as well as significance of permutation tests in Markov random field module score, function consistency score and Pearson correlation coefficient difference score. Three breast cancer risk modules were identified from a breast cancer-related interaction network. Genes in these risk modules were confirmed to play critical roles in breast cancer by literature review. These risk modules were enriched in breast cancer-related pathways or functions and could distinguish between breast tumor and normal samples with high accuracy for not only the microarray dataset used for breast cancer risk module identification, but also another two independent datasets. Our integrated strategy could be extended to other complex diseases to identify their risk modules and reveal their pathogenesis.

## INTRODUCTION

Breast cancer is one of leading causes of cancer death for females worldwide [1]. The morbidity of breast cancer continues to rise over the past few decades, which makes breast cancer an increasing global health issue.

Complex diseases, including breast cancer, are not caused by a single gene mutation. Instead, they are the consequence of multifaceted dysfunctions, including protein/coding genes, non-coding RNAs and their epigenetic modification [2, 3]. Disease risks of involved genes spread along the links of related biological networks since most genes execute their cellular functions by interacting with other genes [4, 5]. Disease-associated genes do not scatter randomly in networks, but tend to interact with each other. A tightly clustered subgraph of disease-associated genes from the same network neighborhood forms a disease risk module with more internal connections than expected randomly in the whole network [6]. Previous studies have shown that within a disease risk module, genes present significant similarities in terms of expression, function and disease association for complex diseases, such as cancers and cardiovascular diseases [7, 8]. Therefore, the identification of disease risk modules could contribute to understanding the molecular mechanisms underlying breast cancer.

Disease risk modules could be detected directly from undirected networks, i.e. gene/protein interaction networks and co-expression networks, by available tools. For example, Cytoscape plugin MCODE has been applied to disease risk module detection from interaction networks for lung adenocarcinoma [9], non-small-cell lung cancer [10], colorectal cancer [11] and inflammatory bowel diseases [12]. In co-expression networks, disease risk modules were detected using GraphWeb for pancreatic ductal adenocarcinoma [13], or by weighted gene co-expression network analysis (WGCNA) for atopic dermatitis [14] and Alzheimer’s disease [15].

Disease risk modules could also be detected by merging or extending cliques. In graph theory, a clique in a network is a fully connected subgraph where every two nodes are connected by an edge [16]. Cliques with disease-related genes are associated with complex diseases and are of great value to uncovering disease pathogenesis since perturbation of any gene in a clique will directly destroy the function of its neighbors. Disease risk modules were detected by merging cliques highly overlapped for ankylosing spondylitis [17], congenital heart defects in Down syndrome [18] and narcolepsy [19], or by extending cliques for multiple diseases [20].

**Figure 1:**
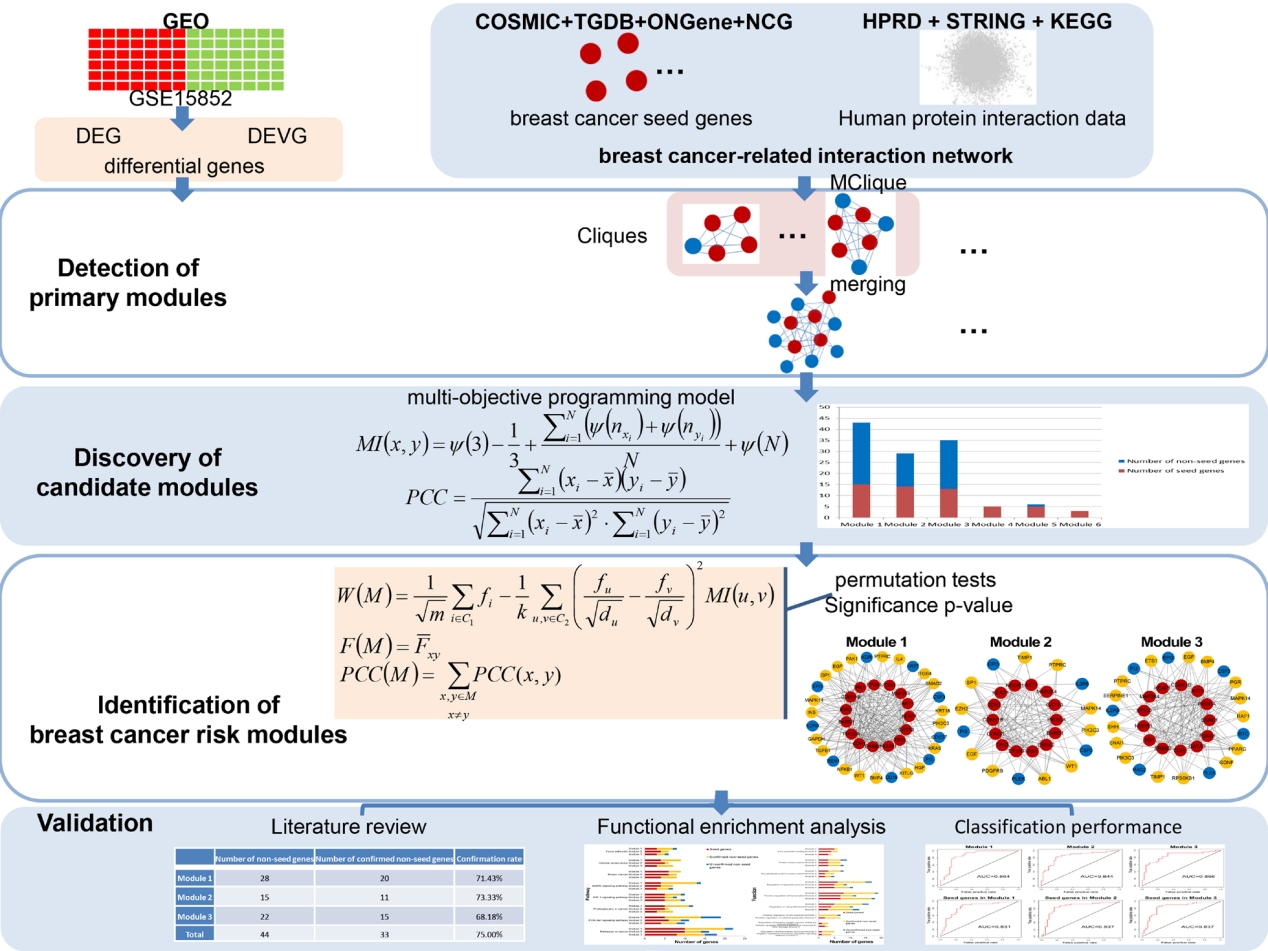
**A schematic diagram of the integrated breast cancer risk module identification strategy.**

Mathematical programming has been used to solve network reconstruction problems to achieving globally optimal number of edges and number of quantitative differences [21]. Such kind of programming could also be applied to module identification for optimal disease association. Thus, in this paper, according to a multi-objective programming model for two similarity criteria maximization as well as significance in module score based on Markov random field (MRF), consistency score of functions and difference score for Pearson correlation coefficient (PCC), an integrated disease risk module identification strategy was proposed to identify breast cancer risk modules from a breast cancer-related interaction network. The association of these risk modules with breast cancer was validated by confirmation rate of literature review, functional enrichment analysis, and classification accuracy (Figure 1).

## RESULTS

### Primary modules

After evaluating differential information of genes with our two measurements (see Materials and Methods), 218 differentially expressed genes (DEGs) and 1209 differential expression variance genes (DEVGs) were obtained. Hence, 1382 differential genes were screened out from the microarray dataset.

161 cliques containing DEGs or DEVGs with >4 nodes were mined from the breast cancer-related interaction network. After merging cliques/subgraphs with extent of overlapping for seed genes in different cliques *S* > 0.8 (see Materials and Methods), 6 primary modules were discovered (Table 1).

**Table 1:** Primary modules.

	**Number of genes**	**Number of seed genes**	**Number of non-seed genes**
Primary module 1	91	15	76
Primary module 2	61	14	47
Primary module 3	59	13	46
Primary module 4	6	5	1
Primary module 5	7	5	2
Primary module 6	4	3	1

### Candidate modules

**Figure 2:**
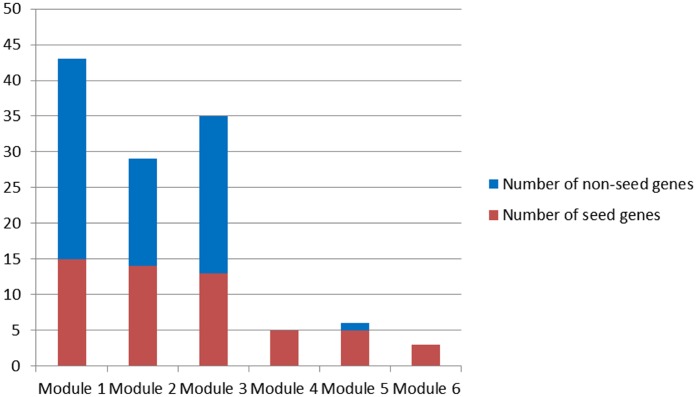
**Candidate modules.**

Corresponding to primary modules, 6 candidate modules were discovered based on two criteria maximization using a multi-objective programming model (see Materials and Methods, Figure 2).

Since only seed genes were contained in Module 4 and 6, Module 1-3 and 5 were candidate modules for breast cancer risk module identification.

### Breast cancer risk modules

**Figure 3:**
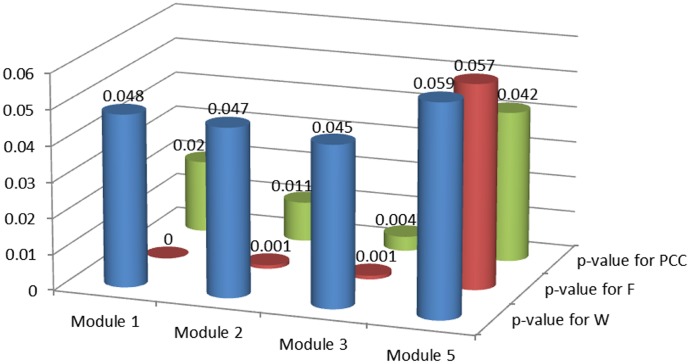
**P-values of permutation tests for candidate modules.**

**Figure 4:**
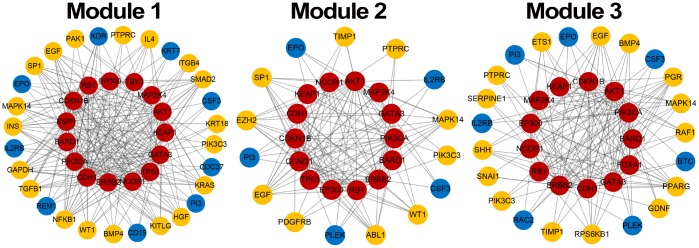
**Breast cancer risk modules.** Red nodes are seed genes, yellow are confirmed non-seed genes and blue are unconfirmed non-seed genes.

Module score *W* based on MRF, consistency score *F* of functions and difference score for PCC were calculated for candidate modules and 1,000 random modules with the same number of genes. According to the significance of permutation tests for candidate modules (see Materials and Methods), 3 breast cancer risk modules were identified (Figure 3) involving 16 seed genes and 44 non-seed genes (Figure 4). 8 non-seed genes were in all three breast cancer risk modules.

### Validation of breast cancer risk modules

The association of these risk modules with breast cancer was evaluated during the validation process from three aspects (see Materials and Methods).

### Literature review

A literature review was carried out using the online database PubMed (https://www.ncbi.nlm.nih.gov/pubmed) for non-seed genes in 3 breast cancer risk modules. It was showed that 75% (33/44) non-seed genes were associated with breast cancer (~70% for each module, Table 2), demonstrating disease association of risk modules identified by our integrated strategy.

**Table 2:** The confirmation rate of non-seed genes in breast cancer risk modules.

	**Number of non-seed genes**	**Number of confirmed non-seed genes**	**Confirmation rate**
Module 1	28	20	71.43%
Module 2	15	11	73.33%
Module 3	22	15	68.18%
Total	44	33	75.00%

In literature, 5 of 8 common non-seed genes in all three breast cancer risk modules were verified. Ling et al. found that the mRNA expression level of PIK3C3 was steadily increased during breast cancer progression and elevated at stage IV [22]. MAPK14, as one of the hub target genes in a PPI network constructed by Wang et al., had the potential to be used as candidate targets for breast cancer treatment [23].

For other non-seed genes in breast cancer risk modules, a transcriptomic signature of BMP4 signaling exhibited by primary ER^+^ breast tumor patients was predictive of improved disease outcome or an improved biologic response to the treatment. This highlighted BMP4 and its downstream pathway activation as a therapeutic opportunity in ER^+^ breast cancer [24]. Considerable evidence has implicated WT1 in the development, pathogenesis and therapy of breast cancer [25]. For example, WT1 expression levels in breast cancers were significantly higher than in control tissue [26]. WT1 has also been linked with in breast cancer malignant transformation, and its overexpression associated with reduced susceptibility to drug treatment [27]. ETS1 has versatile roles during the cellular processes of various types of cancers. It was often highly expressed in breast cancer samples and mediated migration and invasion of human breast cancer cells [28].

These non-seed genes in our risk modules played vital roles in the development, pathogenesis or signaling of breast cancer. Therefore, these genes could act as potential breast cancer genes.

### Functional enrichment analysis

To assess the functional information of genes in breast cancer risk modules, genes in each module were tested for enrichment against KEGG pathways and GO functions (GO-terms of biological process and molecular function) using the Enrichr tool, respectively.

**Figure 5:**
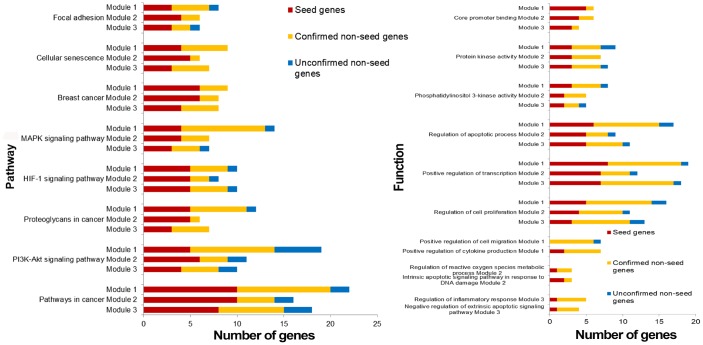
**Pathways and functions enriched by breast cancer risk modules.**

**Figure 6:**
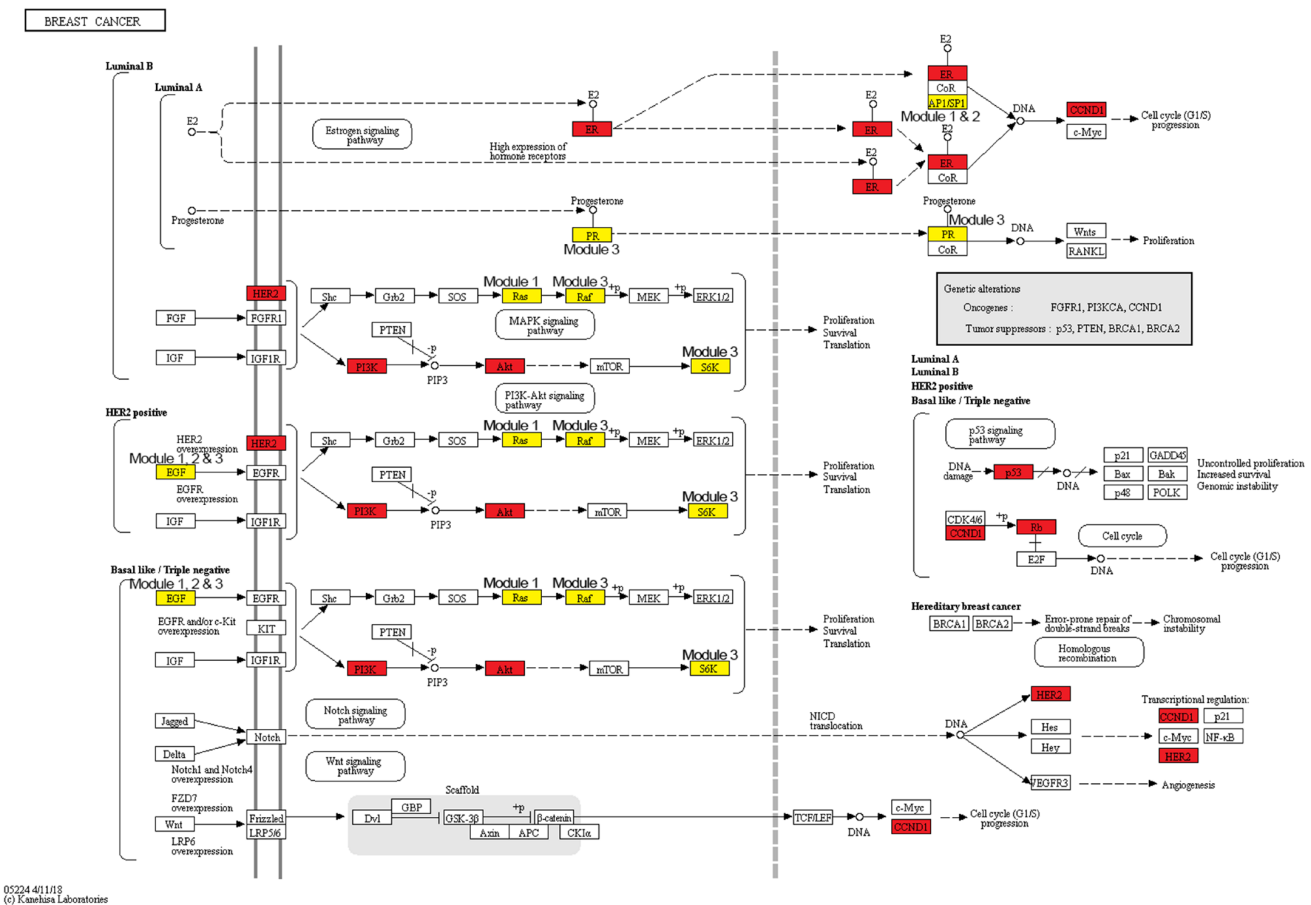
**The breast cancer pathway. Red nodes are seed genes and yellow are non-seed genes.** Modules these genes belong to are marked beside them.

Breast cancer risk modules were enriched in numerous pathways and functions, especially breast cancer-related ones (Figure 5). It was worth noting that genes from all three risk modules, including seed genes and non-seed genes, were enriched in the “breast cancer” pathway (Figure 6), indicating their roles in the process of breast cancer. Different locations of genes from different modules demonstrated various functions of modules.

Other pathways and functions enriched by single breast cancer risk module or all risk modules were also closely associated with breast cancer. “Positive regulation of cell migration” was also a breast cancer-related biological process, which was enriched by only non-seed genes in Module 1. Bisphosphonates, antiresorptive drugs, might be developed as a therapeutic option for breast cancer since it significantly decreased cancer cell migration in a dose-dependent manner [29]. Oxidative stress, the process of oxidative damage caused by Module-2-enriched function “Regulation of reactive oxygen species metabolic process” [30], and “Regulation of inflammatory response” enriched by genes in Module 3, were both associated with breast cancer development [31].

For functions enriched by all risk modules, “PI3K-Akt signaling pathway” is an important signal transduction pathway in cells, which was closely associated with the lymph node metastasis of breast cancer, and could affect breast cancer progression and patient prognosis [32, 33]. “Cellular senescence” is a complex process that was found to be a tumor-suppressive mechanism leading to suppressed breast cancer cell proliferation by inhibiting cell proliferation [34, 35]. “Positive regulation of transcription” played significant roles in breast cancer development since it was the function enriched in by genes identified from many breast cancer-related researches [36]. It was also revealed that via participating in regulation of transcription biological processes, biological elements were involved in the progression of breast cancer [37]. An aberrant apoptotic process can lead to several pathological conditions, such as tumorigenesis and cancer metastasis [38]. Thus, mediating through active “regulation of the apoptotic process”, drugs could effect on breast cancer cells [39]. DNA-dependent protein kinase has an important role with DNA double-strand break repair. DNA-dependent “protein kinase activity” of peripheral blood lymphocytes is associated with risk of breast cancer since the activity in breast cancer patients was significantly lower than that in normal [40].

Most genes in our breast cancer risk modules, especially non-seed genes, were enriched in breast cancer-related pathways or functions, some of which were also related to cancer hallmark-associated GO terms, such as “HALLMARK_APOPTOSIS” and “HALLMARK_PI3K_AKT_MTOR_SIGNALING”, which indicated the disease association of our risk modules.

### Classification performance

With genes in breast cancer risk modules as features, breast tumor and normal samples were classified by the SVM classifier. Using LOOCV, all three risk modules achieved an accuracy of approximate 85% for breast tumor and normal samples of GSE15852, although only ~33% genes in each module were differential genes.

**Figure 7:**
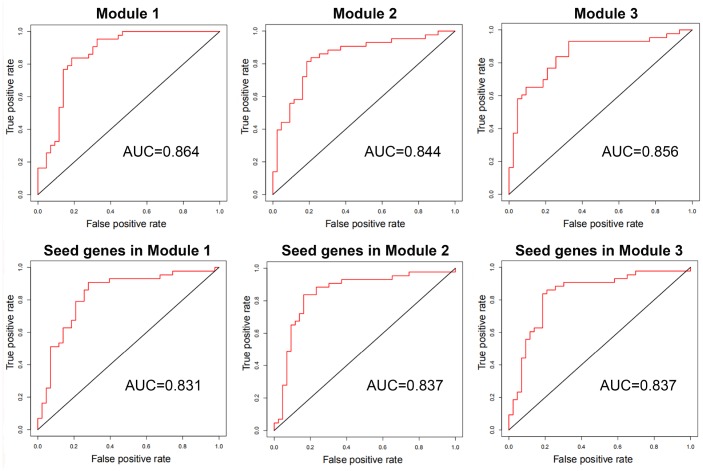
**The ROC curves and AUC values of breast cancer risk modules and seed genes in these modules for GSE15852.**

In order to compare the classification performance of breast cancer risk modules and that of only seed genes in these modules, the classification accuracy was also computed based on SVM classifier with seed genes as features. The classification accuracy was ~83% for seed genes in each risk module, which was inferior to that of breast cancer risk modules (Figure 7).

These results exhibited that our risk modules with seed genes and non-seed genes could distinguish between breast tumor and normal samples with higher accuracy than with only seed genes.

Additionally, to assess classification significance of risk modules, 100 random gene sets of the same size were selected from the breast cancer-related interaction network. AUC values were calculated utilizing SVM classifier with genes of random gene sets as features. The classification accuracy of breast cancer risk modules outperformed the accuracy of random gene sets of the same size (AUC = ~0.82). This showed that risk modules could classify breast tumor and normal samples effectively with significantly better performance than only seed genes or random selected ones.

Then, the classification with genes in breast cancer risk modules as features was conducted on another two datasets, GSE70947 from another platform and samples collected from The Cancer Genome Atlas (TCGA), the accuracy of which was compare with that of seed genes in risk modules (Table 3). Since the size of breast tumor in TCGA was much larger than that of normal samples, tumor samples with the same number as normal ones (113) were randomly selected. The genes in breast cancer risk modules could also classify breast tumor and normal samples of the same size accurately (>0.86).

**Table 3:** The classification accuracy with breast cancer risk modules and seed genes in risk modules as features for another two datasets.

	**Microarray dataset GSE70947**			**Expression data from TCGA**		
	**Module 1**	**Module 2**	**Module 3**	**Module 1**	**Module 2**	**Module 3**
AUC values with breast cancer risk modules	0.899	0.893	0.889	0.985	0.989	0.992
AUC values with seed genes in risk modules	0.804	0.778	0.783	0.974	0.976	0.977

Similar results that our risk modules with both seed genes and non-seed genes could distinguish between breast tumor and normal samples with higher accuracy than with only seed genes were also obtained for these two datasets.

## DISCUSSION

In this paper, an integrated strategy was proposed to identify breast cancer risk modules according to a multi-objective programming model and significance in three scores. A total of 3 breast cancer risk modules were identified. ~70% non-seed genes in these risk modules were confirmed to play vital roles in the development, pathogenesis or signaling of breast cancer and could act as potential breast cancer genes. Most genes in our risk modules, including unconfirmed non-seed genes, were enriched in breast cancer-related pathways or functions. These risk modules could distinguish between breast tumor and normal samples with higher accuracy than seed genes in risk modules. These results indicated the disease association of breast cancer risk modules identified by our integrated strategy.

**Figure 8:**
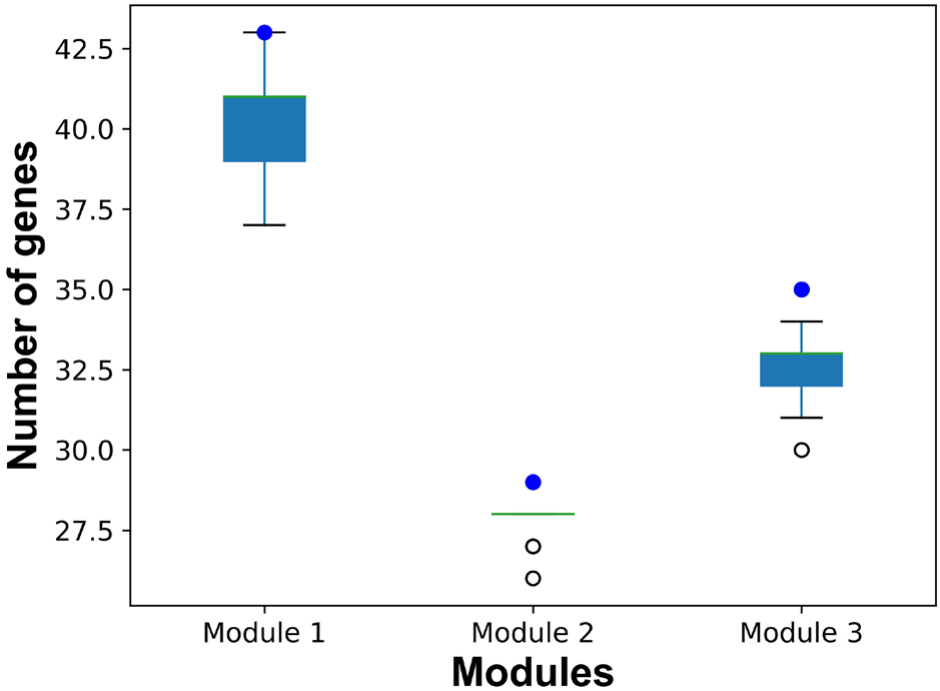
**The number of common genes in risk modules from all samples and from random samples.** Blue dots represent the number of genes in breast cancer risk modules from all samples. Boxplots represent the distribution of the number of common genes in breast cancer risk modules from all samples and risk modules form random samples.

In order to illustrate the robustness of our risk modules, risk modules were re-identified using 90% samples randomly selected from GSE15852. The process was repeated 100 times. Genes of risk modules from random samples were compared with those of breast cancer risk modules from all samples (Figure 8). More than 90% genes in risk modules from all samples were re-identified by random samples.

**Figure 9:**
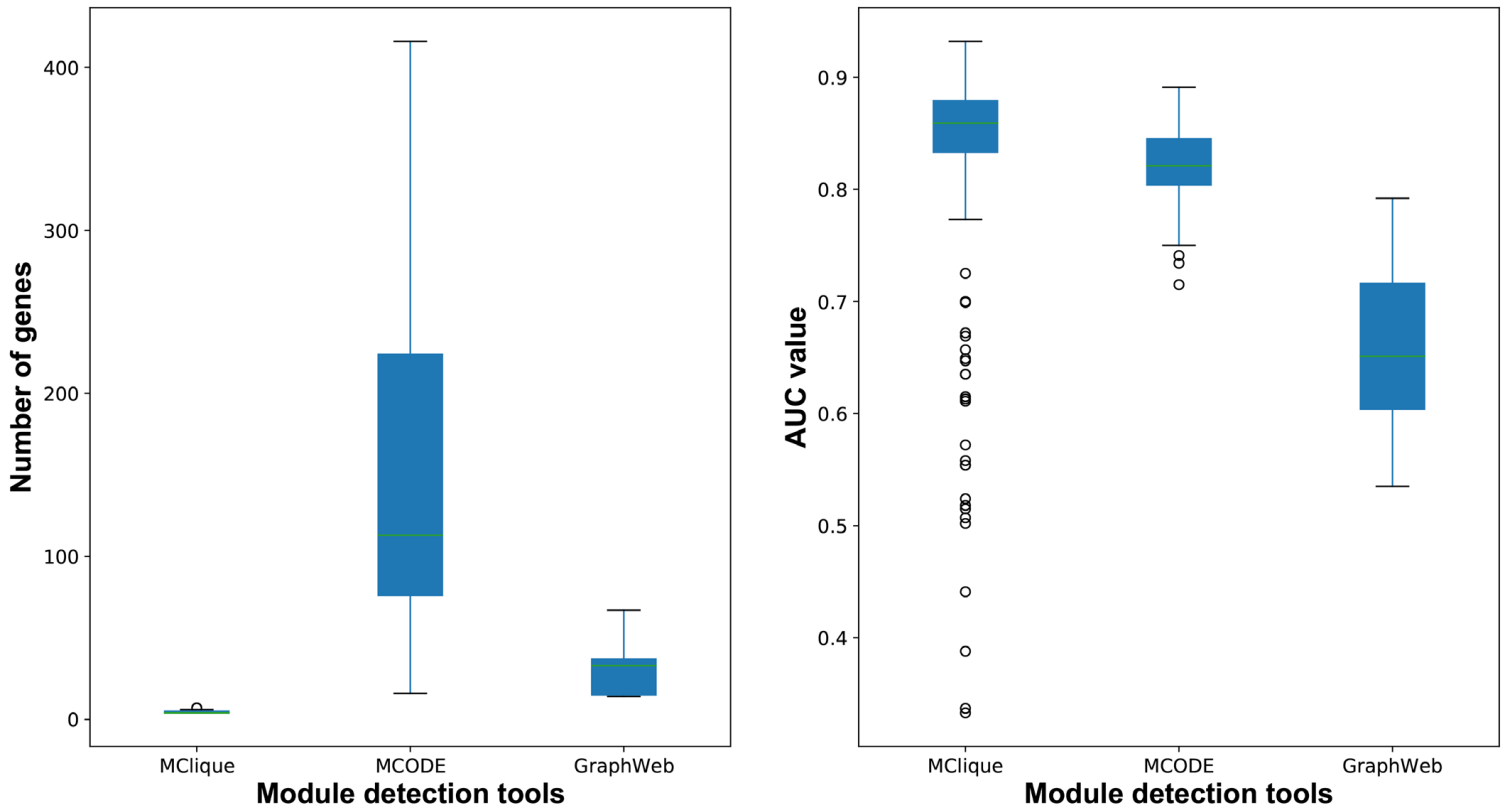
**The number of genes and classification accuracy for cliques/modules detected by MClique, MCODE and GraphWeb.**

For the purpose of primary modules detection, cliques of the breast cancer-related interaction network were mined using Cytoscape MClique in our integrated strategy. MCODE and GraphWeb were also taken into consideration for primary module detection. It was showed that the number of genes in cliques/modules mined by MClique, MCODE and GraphWeb had great difference, while classification accuracy varied among the three module sets (Figure 9). Since cliques mined by MClique were smaller with more connections, larger AUC values and more similar genes, they were used for primary module detection in our integrated strategy.

**Figure 10:**
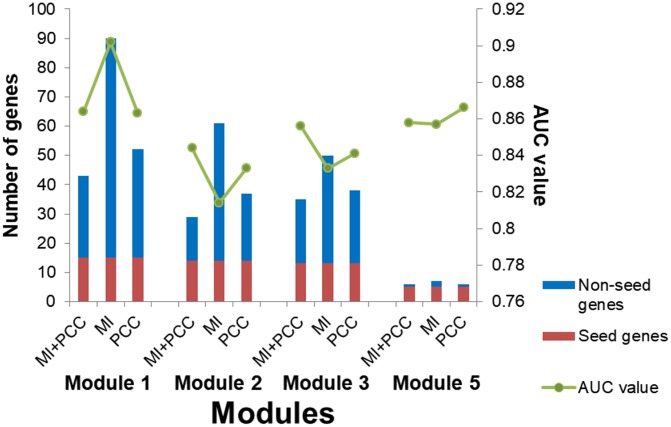
**The number of genes and AUC values for candidate modules discovered using different criteria.**

A multi-objective programming model based on maximization of two criteria, MI and PCC, was employed for candidate module discovery. Candidate modules based on individual criterion, MI or PCC, were also discovered. Breast tumor and normal samples were classified with these modules as features (Figure 10). Candidate modules discovered using both criteria could classify samples with higher accuracy and fewer genes than those using individual criterion in most cases, and were used for breast cancer risk module identification.

To further demonstrate the effectiveness of our integrated strategy, genes removed from primary modules were compared to non-seed genes remained in breast cancer risk modules by classification accuracy. The accuracy of non-seed genes remained in breast cancer risk modules was higher than that of genes removed from primary modules (Table 4). These results indicated that non-seed genes in breast cancer risk modules were more related to breast cancer than the removed ones, supporting the effectiveness of our integrated strategy, especially the multi-objective programming model.

**Table 4:** The classification accuracy of genes removed from primary modules and non-seed genes remained in breast cancer risk modules.

	**Genes removed from primary modules**	**Non-seed genes remained in breast cancer risk modules**
Module 1	0.856	0.889
Module 2	0.795	0.878
Module 3	0.847	0.908

Results of breast cancer risk modules and their validation demonstrated the effectiveness of our choice for different steps of our integrated strategy. Nevertheless, there are some potential limitations to our study. First, known disease-associated genes were required for disease-related interaction network construction. Second, expression similarity and difference as well as functions of genes were exploited for disease risk module identification in our integrated strategy, which might be affected by datasets used in the process. The other breast cancer microarray dataset GSE70947 was used to screen differential genes and identify breast cancer risk modules with our integrated strategy. Another two risk modules were identified. With more differential genes in these modules (~55%), they had a high classification accuracy (~0.85), which was still inferior to that of our risk modules identified from GSE15852 (>0.89) for GSE70947. This showed the effectiveness of our integrated strategy and breast cancer risk modules we identified. With other types of information available, they could also be integrated into our integrated strategy to improve its performance or reveal the molecular mechanism of metastasis [41].

Subtype (Basal, Her2, Luminal A (LumA) and Luminal B (LumB)) classification for breast cancer is of great significance for its clinical diagnosis and treatment. To assess the subtype classification ability of breast cancer risk modules, expression values of genes in these risk modules were used to classify different subtypes. All risk modules could distinguish between subtypes with high accuracy (Table 5).

**Table 5:** The classification accuracy of breast cancer risk modules for breast cancer subtypes.

	**Module 1**	**Module 2**	**Module 3**
Basal vs Her2	0.978	0.964	0.985
Basal vs LumA	0.999	0.999	0.996
Basal vs LumB	0.995	0.990	0.991
Her2 vs LumA	0.978	0.983	0.972
Her2 vs LumB	0.938	0.913	0.902
LumA vs LumB	0.845	0.863	0.842

In summary, breast cancer risk modules identified by our integrated strategy were confirmed to play critical roles in breast cancer by literature review, functional enrichment analysis, and classification accuracy. Our integrated breast cancer risk module identification strategy could be extended to other complex diseases for researchers to gain more thorough understanding of their pathogenesis.

## MATERIALS AND METHODS

### Data

The microarray dataset GSE15852 (GPL96) was downloaded from Gene Expression Omnibus (GEO) database, which was composed of 43 human breast tumor tissues and their 43 paired normal tissues.

Known breast cancer-associated genes were collected from the Catalogue of Somatic Mutations in Cancer (COSMIC) Cancer Gene Census (CGC, https://cancer.sanger.ac.uk/census), the breast cancer gene database of the Tumor Gene Family of Databases (TGDB, http://www.tumor-gene.org/tgdf.html), ONGene (http://www.ongene.bioinfo-minzhao.org/) and the Network of Cancer Genes (NCG, http://ncg.kcl.ac.uk/index.php). CGC is an expert-curated and wide-used source of genes driving human cancer [42]. TGDB contains information about genes which are targets for cancer-causing mutations with their historical relevance [43]. ONGene is a literature-based database for human oncogenes [44]. The latest version of NCG contains information of cancer genes from manually curated publications [45]. 32 breast cancer-associated genes from at least two databases were referred to as seed genes in our analysis to increase the confidence of our seed genes (Table 6).

**Table 6:** Breast cancer-associated genes and their source databases.

	**CGC**	**TGDB**	**ONGene**	**NCG**
BARD1	√		√	
BRCA1	√	√		√
BRCA2	√	√		√
RB1	√	√		√
TP53	√	√		√
AKT1	√		√	√
ARID1A	√			√
ARID1B	√			√
BAP1	√			√
CASP8	√			√
CCND1	√	√	√	√
CDH1	√		√	√
CDKN1B	√		√	√
CTCF	√			√
EP300	√			√
ERBB2	√	√	√	√
ESR1	√	√		√
FOXA1	√			√
GATA3	√			√
IRS4	√			√
MAP2K4	√			√
MAP3K1	√			√
MAP3K13	√			√
NCOR1	√			√
NOTCH1	√			√
NTRK3	√			√
PBRM1	√			√
PIK3CA	√		√	√
PPM1D	√		√	
SMARCD1	√			√
TBX3	√			√
ZMYM3	√			√

A complete gene/protein interaction network is of fundamental importance for the understanding of diseases [46]. Human protein interaction data were integrated from the HPRD [47], STRING [48], and KEGG [49] databases. All products of seed genes were used to determine a breast cancer-related interaction network by extracting direct interactions between seed and other proteins. The resulting network was centered on seed genes with 13136 interaction relationships between 5202 genes.

### Breast cancer risk module identification strategy

An integrated disease risk module identification strategy was proposed and used to identify breast cancer risk modules. First, differential genes were screened from a breast cancer microarray dataset, and primary modules were detected by merging cliques containing differential genes from a breast cancer-related interaction network. Then, candidate modules were discovered using a multi-objective programming model to maximize two similarity criteria. Finally, breast cancer risk modules were identified according to significance in module score based on MRF, consistency score of functions and difference score for PCC.

### Detection of primary modules

Two measurements were used to evaluate differential information of genes: (1) after preprocessing, the significance analysis of microarrays (SAM) program was used for screening DEGs. The false discovery rate (FDR) < 0.05 and the absolute value of log_2_fold change (FC) > 1 were selected as the significance threshold for DEG screening. (2) The variance *S*^2^ for expression values of gene *x* could measure how far its expression level in different samples is from the average expression level in all samples:

$$S^{2}=\frac{\sum_{i=1}^{N}{\left(x_{i}-\bar{x}\right)}^{2}}{N-1}$$ (1)

where $\left(x_{1},\cdots ,x_{N}\right)$ represents expression value of gene *x* (*N* is the number of samples), $\bar{x}$ is the average value for $\left(x_{1},\cdots ,x_{N}\right)$. The variation value *V* was defined as the absolute value of difference between variance for genes in disease and normal samples:

$$V=abs\left(S_{normal}^{2}-S_{tumor}^{2}\right).$$ (2)

The p value was evaluated by the number of times *V* of 1000 random genes exceed that of the interested one. Genes with FDR-adjusted p value<0.05 were screened as DEVGs.

DEGs and DEVGs were differential genes for further analysis.

Cliques containing 4-8 genes were mined from the breast cancer-related interaction network using Cytoscape MClique. Only those containing differential genes were taken into consideration. These cliques were also centered on seed genes that overlapped with seed genes in other cliques. The extent of overlapping for seed genes in different cliques was evaluated by Simpson index:

$$S\left(A,B\right)=\frac{\left|A\cap B\right|}{\min\left(\left|A\right|,\left|B\right|\right)}$$ (3)

where A∩B is common genes in cliques *A* and *B*, |A| and |B| indicate the number of genes in module *A* and *B*, respectively.

Cliques with 4 genes could be merged if the cutoff of Simpson index *S* was set to ~0.75. In this case, subgraphs with more than 2000 genes were obtained. Therefore, to identify more rational modules, cliques with 5-8 genes were used for clique merging. That is, each pair of cliques/subgraphs with *S*>0.8 were merged to form larger subgraphs until *S* for no subgraph pair was larger than 0.8. Subgraphs obtained at this step were named primary modules.

### Discovery of candidate modules

Using a multi-objective programming model to maximize two criteria, candidate modules were discovered, under the hypothesis that genes more similar to seed genes may tend to be disease-related. The similarity of non-seed genes with seed ones in primary modules was measured by the sum of mutual information (MI) and the average of PCCs:

$$\max\sum MI\left(x,y\right),\bar{PCC}\left(x,y\right)$$ (4)

Subject to:

$$\begin{array}{l} MI\left(x,y\right)=\psi \left(3\right)-\frac{1}{3}+\frac{\sum_{i=1}^{N}\left(\psi \left(n_{x_{i}}\right)+\psi \left(n_{y_{i}}\right)\right)}{N}+\psi \left(N\right)\\ PCC=\frac{\sum_{i=1}^{N}\left(x_{i}-\bar{x}\right)\left(y_{i}-\bar{y}\right)}{\sqrt{\sum_{i=1}^{N}{\left(x_{i}-\bar{x}\right)}^{2}\cdot \sum_{i=1}^{N}{\left(y_{i}-\bar{y}\right)}^{2}}}\\ x,y\in \text{primary}\;\text{module}\\ x\in \text{seed}\;\text{genes}\\ y\notin \text{seed}\;\text{genes} \end{array}$$

where $n_{x_{i}}$ and $n_{y_{i}}$ are the number of points within a certain radius determined by 3 nearest neighbors of $\left(x_{i},y_{i}\right)$, and the digamma function $\psi \left(t\right)=\Gamma {\left(t\right)}^{-1}d\Gamma \left(t\right)/dt$.

Through multiple iterations that calculations were performed for each non-seed gene *y* against each seed gene *x* in primary modules, candidate modules that satisfied the requirements could be obtained.

### Identification of breast cancer risk modules

Breast cancer risk modules were identified based on significance of permutation tests for three scores of candidate modules.

### Module score W based on MRF

For a candidate module with *m* genes, a multivariate random variable $f=\left(f_{1},\cdots ,f_{m}\right)$ was defined as the expression difference of these genes between tumor and normal samples. It was assumed that the expression difference formed a MRF, and thus, the expression difference of a gene only depended on the expression difference of its direct interacting neighbor genes.

Gibbs distribution was employed to specify the joint probability of *f*:

$$P\left(f\right)=\frac{1}{K}e^{-\frac{1}{T}G\left(f\right)}$$ (5)

where *K* is a constant that guarantees the probability sum to be 1, *T* is a temperature parameter controlling the distribution sharpness, and

$$G\left(f\right)=-\frac{1}{\sqrt{m}}\sum_{i\in C_{1}}f_{i}+\frac{1}{k}\sum_{i,j\in C_{2}}{\left(\frac{f_{i}}{\sqrt{d_{i}}}-\frac{f_{i}}{\sqrt{d_{j}}}\right)}^{2}MI\left(i,j\right),$$ (6)

which represents the differential level of seed genes with the similarity between non-seed genes of a candidate module.

Therefore, the module score based on MRF was defined as

$$W\left(M\right)=\frac{1}{\sqrt{m}}\sum_{i\in C_{1}}f_{i}-\frac{1}{k}\sum_{u,v\in C_{2}}{\left(\frac{f_{u}}{\sqrt{d_{u}}}-\frac{f_{v}}{\sqrt{d_{v}}}\right)}^{2}MI\left(u,v\right)$$ (7)

where *k* is the number of interactions in the module *M*, *C*_1_ and *C*_2_ are the set of seed genes and non-seed genes in the module, *f_u_* and *f_v_* are expression differences assessed by the t-test between tumor and normal samples, and *d_u_* and *d_v_* are the degree of non-seed genes *u* and *v*, respectively.

### Consistency score F of functions

The consistency scores of candidate modules were calculated based on functional consistency between seed genes and non-seed ones:

$$F(M)={\bar{F}}_{xy}$$ (8)

where $F_{xy}=\frac{\left|F_{x}\cap F_{y}\right|}{\left|F_{x}\cup F_{y}\right|}$. *F_x_* and *F_y_* are functions annotated by seed gene *x* and non-seed gene *y*, respectively. $\left|F_{x}\cap F_{y}\right|$ is the number of common functions, and $\left|F_{x}\cup F_{y}\right|$ is the number of all functions *x* and *y* annotated.

### Difference score for PCC

PCC between each gene pair in candidate modules for tumor and normal samples were calculated, respectively. PCC difference of a module was defined as the sum of PCC differences for gene pairs in it.

$$PCC\left(M\right)=\sum_{\begin{array}{c} x,y\in M\\ x\neq y \end{array}}PCC\left(x,y\right)$$ (9)

Candidate modules with large values for all the three scores indicated their disease association.

To obtain the significance of permutation tests for each candidate module, 1,000 random modules with the same number of genes were constructed. All of the three scores were calculated individually for these random modules. Scores significantly greater than the random ones (permutation tests, p <0.05) were considered significant. Breast cancer risk modules were identified as candidate modules significant in all 3 scores.

### Validation of breast cancer risk modules

Validation for association of risk modules identified using our integrated strategy with breast cancer was evaluated from three aspects: 1) confirmation rate by literature review, 2) functional enrichment analysis (adjusted p < 0.05 was considered statistically significant), including Gene Ontology (GO) function and Kyoto Encyclopedia of Genes and Genomes (KEGG) pathway analysis, performed through a latest enrichment tool Enrichr (http://amp.pharm.mssm.edu/Enrichr/) [50], and 3) classification accuracy determined by area under the receiver operating characteristic curve (AUC) evaluated with a leave-one-out cross-validation (LOOCV) strategy after distinguishing breast tumor and normal samples by means of a support vector machine (SVM) classifier with genes in risk modules as features. The classification was conducted on not only the microarray dataset we used to identify breast cancer risk modules, but also another two independent datasets: one was another microarray dataset GSE70947 (GPL13607) downloaded from GEO composed of 148 human breast tumor tissues and 148 paired adjacent normal breast tissue, and the other was the expression data of 1102 breast tumor and 113 normal samples collected from the TCGA database (https://portal.gdc.cancer.gov/) [51].

## AUTHORS CONTRIBUTIONS

W.L., G.D., J.Z., and L.C. contributed to the study design. G.D. and J.Z. contributed to data collection. W.L., G.D., and J.Z. performed statistical analysis, interpretation, and drafted the manuscript. E.H., Y.H., J.L., X.S., K.W., and L.C. revised the manuscript. All authors contributed to critical revision of the final manuscript and approved the final version of the manuscript. W.L., X.S., K.W., and L.C. provided financial support and study supervision.
